# Bamboo Charcoal/Poly(L-lactide) Fiber Webs Prepared Using Laser-Heated Melt Electrospinning

**DOI:** 10.3390/polym13162776

**Published:** 2021-08-18

**Authors:** Zongzi Hou, Nahoko Itagaki, Haruki Kobayashi, Katsufumi Tanaka, Wataru Takarada, Takeshi Kikutani, Midori Takasaki

**Affiliations:** 1 Doctoral Program of Materials Chemistry, Graduate School of Science and Technology, Kyoto Institute of Technology, Kyoto 606-8585, Japan; d9871502@edu.kit.ac.jp; 2 Undergraduate Program of Materials Science, Faculty of Science and Technology, Kyoto Institute of Technology, Kyoto 606-8585, Japan; hoihoi5269@gmail.com; 3 Faculty of Materials Science and Engineering, Kyoto Institute of Technology, Kyoto 606-8585, Japan; haruki@kit.ac.jp (H.K.); ktanaka@kit.ac.jp (K.T.); 4 Department of Materials Science and Engineering, Tokyo Institute of Technology, 2-12-1 Ookayama, Meguro-ku, Tokyo 152-8550, Japan; takarada.w.aa@m.titech.ac.jp; 5 School of Materials and Chemical Technology, Tokyo Institute of Technology, 4259-J3-142, Nagatsuta-cho, Midori-ku, Yokohama 226-8503, Japan; kikutani.t.aa@m.titech.ac.jp

**Keywords:** polylactide, bamboo charcoal, melt-electrospinning, ultrafine fibers, UV shielding, carbon dioxide laser

## Abstract

Although several studies have reported that the addition of bamboo charcoal (BC) to polylactide (PLA) enhances the properties of PLA, to date, no study has been reported on the fabrication of ultrafine BC/poly(L-lactide) (PLLA) webs via electrospinning. Therefore, ultrafine fiber webs of PLLA and BC/PLLA were prepared using PLLA and BC/PLLA raw fibers via a novel laser electrospinning method. Ultrafine PLLA and BC/PLLA fibers with average diameters of approximately 1 μm and coefficients of variation of 13–23 and 20–46% were obtained. Via wide-angle X-ray diffraction (WAXD) analysis, highly oriented crystals were detected in the raw fibers; however, WAXD patterns of both PLLA and BC/PLLA webs implied an amorphous structure of PLLA. Polarizing microscopy images revealed that the webs comprised ultrafine fibers with uniform diameters and wide variations in birefringence. Temperature-modulated differential scanning calorimetry measurements indicated that the degree of order of the crystals in the fibers was lower and the molecules in the fibers had higher mobilities than those in the raw fibers. Transmittance of BC/PLLA webs with an area density of 2.6 mg/cm^2^ suggested that the addition of BC improved UV-shielding efficiencies.

## 1. Introduction

Ultrafine fibers, defined as fibers with diameters in the range from tens of nanometers to a few microns [1,2], have been widely utilized in various applications, including textile and biomedical applications, electronic component fabrication, and environmental purification [3], due to their abundant inter-fiber pores, large surface/volume ratios, excellent flexibilities, and superior adsorption [4,5]. To attain nonwovens with ultrafine fibers, several approaches, such as melt blowing, centrifugal spinning, and electrospinning, have been investigated by numerous researchers [4,6,7,8,9]. Compared with other methods, electrospinning, which can be broadly divided into solution electrospinning and melt electrospinning, is commonly considered to be one of the most economically and technically efficient approaches to obtain ultrafine fibers [5,10]. Solution electrospinning was developed rapidly from a single-fluid blending processes [11] to coaxial [12], side by side [13], tri-axial [14], and other complex processes [15]; however, the high toxicity of organic solvent used in the solution electrospinning for preparation of working fluid is incompatible with the in vivo biomedical applications [16]. Thus, besides convenience and simplicity, energy saving [17] and environmental friendliness [18] are always desired for developing new electrospinning processes. Consequently, an increasing number of researchers have made considerable efforts to produce ultrafine fibers by solvent-free melt electrospinning because of the unavailability of non-toxic accessory substances. On the other hand, one of the most significant limitations of melt electrospinning is the unavoidable thermal degradation. In conventional melt electrospinning, the origin of thermal degradation is attributed to the long residence time of the polymer melt at high temperatures, which were adopted with the aim of decreasing fiber diameters [3,19].

To overcome these limitations, herein, a novel laser electrospinning (LES) method, that is, melt electrospinning with CO_2_ laser irradiation for the rapid heating of polymers, was developed to fabricate ultrafine fibers and substantially suppress the thermal degradation of these fibers. In our previous studies, various ultrafine fibers were successfully produced via LES [20,21]. 

With an increase in global concern for the environment, environmentally friendly and sustainable materials have attracted significant attention as a result of their potential in the realization of the green economy [22,23]. In this regard, polylactide (PLA), which is one of the most commonly used eco-friendly polymers generated from renewable resources including starch from corn or potato, has attracted considerable attention. The properties of crystalline poly-(L-lactide) (PLLA) in PLAs are similar to those of conventional polymers such as polypropylene and polyethylene, and biodegradability with the release of CO_2_ and water as by-products, as well as biocompatibility, are unique properties of PLLA [24]. Studies on ultrafine PLLA fibers obtained by electrospinning have attracted significant interest from many researchers. Zhou et al. [25] fabricated ultrafine PLLA fibers on cellulose fiber filters via melt electrospinning for the collection of sub-micron-sized dust particles. Hong et al. [26] reported the antibacterial activity of polyurethane/poly(lactide-co-glycolide). The dependence of various colorants on the diameter of melt-electrospun fibers in textile applications was discussed by Balakrishnan [3]. 

Recently, the development of ultrafine PLLA fibers blended with particles acquired from natural and biodegradable resources, including bamboo charcoal (BC), has become an interesting topic of research for improving the properties of ultrafine PLLA fibers. For protective applications [27], outdoor protective products with UV-shielding properties have attracted considerable attention because of the limitation of PLLA caused by its high transparency [22,28]. Powder-like BC carbonized from bamboo is a renewable, non-toxic, and antibacterial material with highly conductive, deodorizing, and UV-shielding properties [29,30]. In addition, BC exhibits excellent compatibility with hydrophobic plastic polymers such as PLLA. As reported in previous studies [28], BC/PLLA composites with BC contents from 2.5 wt% to 10 wt% were fabricated successfully. Tensile strength increased with the increasing of BC content until it reached to 7.5 wt%, but decreased with the continuous increase in BC content. UV properties of BC/PLLA was also improved compared with PLLA. BC content higher than 10 wt% resulted in a failure to produce BC/PLLA composite by injection molding. Wang et al. [29] prepared BC/PLA composite with BC contents from 20% to 40% by injection molding and hot press and concluded that the flame retardancy was improved but the flexural properties decreased with the increasing of BC content. These studies indicated that the addition of BC and the increase in BC content resulted in significant effects in terms of the fabrication of BC/PLLA composites with improved properties. 

Although many studies have reported that the addition of BC to PLLA resin enhances the mechanical, thermal, impact resistance, and UV-shielding properties of PLLA [28,31,32], these studies provided us with the basis for research on the enhancement of properties and the widening of applications through the addition of BC into PLLA. In the production of ultrafine fiber, researchers made great efforts to utilize electrospinning methods to produce ultrafine fibers blended with fillers including carbon nanotube [33], nanoclay [34], silver particles, etc. [35]. However, none of them investigated the production of ultrafine fibers with the addition of BC through the electrospinning process; therefore, in order to fill this research gap and expand the application of BC/PLLA products, in our study, LES was conducted to fabricate uniform ultrafine fiber webs of PLLA and BC/PLLA, and the effects of laser power and the addition of BC to the raw fibers, in terms of the thinning behaviors of fibers in these webs during LES, were analyzed. Furthermore, the morphologies, thermal properties, crystalline structures, and UV-shielding properties of the resulting PLLA and BC/PLLA webs were investigated. 

## 2. Materials and Methods

### 2.1. Materials 

The polymer used in this study was PLLA (Nature Works LLC, Minnetonka, MN, USA, melt mass flow rate: 6 g/10 min). The ground BC was blended with PLLA as follows. A BC/PLLA masterbatch with a BC content of 10 wt% was prepared using a twin-screw extruder (KZW20TW-45MG-NH-700, Technovel Co., Ltd., Osaka, Japan) with a rotation speed of 130 rpm at an extrusion temperature of 165 °C.

PLLA and BC/PLLA fibers with 8 wt% BC were produced using the same twin-screw extruder equipped with a spinning nozzle of 2.0 mm diameter. Prior to melt spinning, the PLLA and BC/PLLA fibers were dried at 90 °C for 8 h. Extrusion temperature, screw rotation speed, and mass throughput were set to 165 °C, 60 rpm, and 3 g/min, respectively. The extruded fiber was wound-up on a bobbin at a take-up velocity of 169 m/min. The resultant melt-spun PLLA and BC/PLLA fibers (raw fibers) had average diameters and coefficients of variation (CV) of 164 μm and 9% and 141 μm and 3%, respectively. The raw materials and raw fibers described above were supplied by Shiraishi Biomass Co., Ltd., Kyoto, Japan.

### 2.2. LES

**Figure 1:**
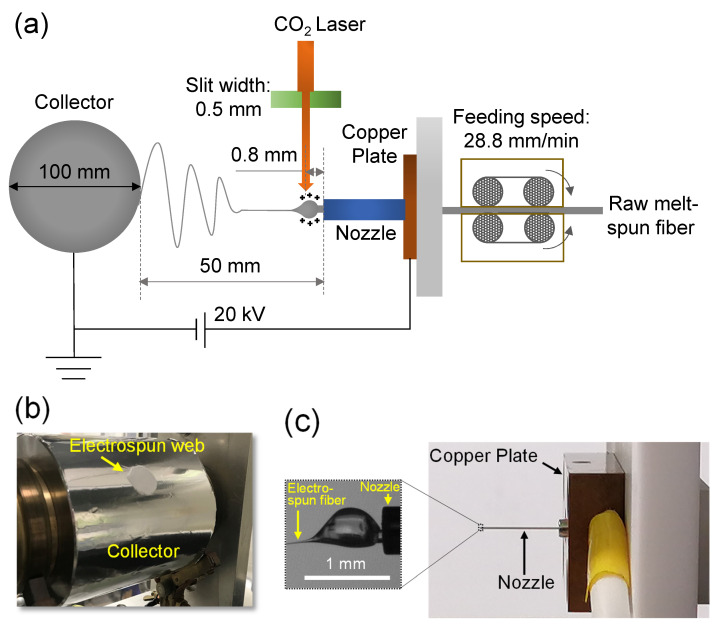
(**a**) Schematic of laser electrospinning (LES), (**b**) image of collector section of LES with web prepared under the stationary state of the collector, and (**c**) image of nozzle section of LES apparatus with an enlarged photograph showing typical spinning behavior.

The LES apparatus used in the present study (Figure 1) was composed of a CO_2_ laser irradiation system (PIN-30R, Onizuka Glass Co., Ltd.; wavelength: 10.6 μm) and an electrospinning system (NEU-010, Katotech Co., Ltd., Kyoto, Japan) [20,21]. The raw fiber was fed by a feed roller at a constant feed rate of 28.8 mm/min through a stainless steel nozzle with inner and outer diameters of 0.31 and 0.57 mm, respectively. The nozzle was connected to a Cu plate to supply a high voltage of 20 kV. The running fiber emerging from the nozzle was rapidly melted by CO_2_ laser irradiation. Subsequently, the molten fiber was driven toward a grounded collecting drum placed 50 mm away from the tip of the nozzle. The LES apparatus was installed in a box, and humidity inside the box was maintained below 40%, delivering dry air flow at a temperature of approximately 25 °C. Considering the effect of the width of the laser beam on the electrospinning behaviors of raw fibers investigated herein [36], the laser beam was deformed to an elliptical shape using a cylindrical lens and cut-off was achieved using a rectangular alumina slit with short and long axes of approximately 0.5 and 4 mm, respectively. The long axis of the slit was perpendicular to the direction of fiber running. The distance between the laser beam axis and the tip of the nozzle was fixed to approximately 800 μm, and the laser power was varied from 6 to 26 W. 

For observation of the electrospinning behaviors of raw fibers and the obtaining of web samples for scanning electron microscopy (SEM), LES was performed using a collector drum with 100 mm diameter and 100 mm width for approximately 4 min. In this case, the collector drum was in a stationary state, that is, rotation or traverse motion was not applied. In contrast, for differential scanning calorimetry (DSC), wide-angle X-ray diffraction (WAXD), and UV shielding measurements, webs with larger masses were acquired by rotating the collector drum for 60 min at a rotation speed of 2.2 m/min and applying traverse motion in the range of approximately 50 mm at a speed of 0.1 m/min.

To observe the fiber spinning behavior during LES, a charge-coupled device (CCD) camera with a telecentric lens of 2× magnification, which was fixed on an adjustable stage, was used. The camera was connected to a computer, and video images were recorded at a rate of 15 fps.

Several sets of a series of still images, with an interval of 1 s, were obtained at various laser powers using an image processing software application (WinROOF 2015, MITANI Corp., Fukui, Japan).

Diameter profiles of the fibers near the nozzle were examined based on the images acquired under each spinning condition. By ignoring the change in fiber density, the fiber running speed (v, (m/s)) at each point along the spinline was calculated from the results of the diameter profiles according to the law of conservation of mass using Equation (1):

(1) $$v={\left(\frac{D_{0}}{D}\right)}^{2}v_{0},$$

where D is the diameter in the spinline (μm), $D_{0}$ is the diameter of the raw melt-spun fiber (μm), and $v_{0}$ is the fiber feeding speed (m/s).

Strain rate ($\dot{\varepsilon }$) profiles were obtained from the investigated diameter profiles (Figure 3) supposing a steady-state process, where $\dot{\varepsilon }$ is presented by

(2) $$\dot{\varepsilon }=\frac{dv}{dx},$$

where x is the distance from the nozzle.

Before analysis, smoothing of the raw data was conducted by applying the moving average operator. 

By integrating the fiber running speed (v) profile along the spinline, the residence time (t(x)) of the material from the tip of the nozzle to a certain distance x during electrospinning was analyzed using Equation (3):

(3) $$t\left(x\right)=\int_{0}^{x}\frac{dx}{v},$$

### 2.3. Fiber Temperature Measurement Using Thermography during LES 

During LES, temperature measurement of the fiber in the vicinity of the melting area was performed in a non-contact mode using infrared radiation thermography (X6540SC, FLIR Systems Inc., Wilsonville, OR, USA) at a spatial resolution of 640 × 512 pixels for the measurement area of 9.6 × 7.7 mm (namely, 150 μm/pixel). From the recorded video image, the fiber temperature was evaluated using ResearchIR software. 

### 2.4. Preparation of Film Samples

Film samples of raw PLLA and BC/PLLA fibers with 8 wt% BC were prepared using a hot press at a compression temperature, pressure, and period of 180 °C, 60 MPa, and 4 min, respectively. A polyimide film with a thickness of 25 μm was employed as a spacer for compression molding. Several polyimide films were stacked to fabricate film samples of various thickness. After molding, the PLLA and BC/PLLA films were removed from the hot press stage and immediately immersed in ice water for instant cooling.

### 2.5. Fourier Transform Infrared (FTIR) Spectroscopy 

To analyze the effectiveness of CO_2_ laser irradiation for heating the raw PLLA and BC/PLLA fibers during LES, absorbances (A) of the PLLA and BC/PLLA films with the thickness ($x_{t}$) of 34, 47, 70, 90, and 111 μm were measured at the CO_2_ laser wavelength of 10.6 μm (wavenumber: 943 cm^−1^) using an FTIR spectrometer (Cary 630, Agilent Technologies, Hachiojishi, Japan). FTIR spectra were acquired under the following conditions: resolution: 2 cm^−1^; wavenumber range: 600–4000 cm^−1^; scans: 64. According to the Beer–Lambert law, the absorption coefficient K can be expressed by Equation (4):

(4) $$A=-\log_{10}T=\frac{K}{2.303}x_{t}+A_{0},$$

where T is the transmittance and $A_{0}$ is the parameter for the effect of surface reflection. From the slope of the A versus $x_{t}$ plot, K was obtained. 

### 2.6. SEM

Images of raw fibers, BC samples, and fibers in the electrospun webs were acquired using a scanning electron microscope (TM3000, Hitachi High-Tech Co., Tokyo, Japan) at an accelerating voltage of 15 kV. Prior to analysis, the samples were fixed on the sample holder using conductive C tape and coated with a layer of Au sprayed by an ion sputter coater (E-1010, Hitachi High-Tech Co., Tokyo, Japan) as a pretreatment. Images of the webs were obtained under various LES conditions. For each image, diameters of the fibers were measured at 200 different positions using a software application (ImageJ version 1.8.0, U. S. National Institutes of Health, Bethesda, MD, USA) to evaluate the average diameter and CV. 

### 2.7. Temperature-Modulated Differential Scanning Calorimetry (TMDSC)

TMDSC was executed using a differential scanning calorimeter (DSC-60A Plus, Shimadzu Co., Kyoto, Japan). Approximately 10 and 2 mg powder-like raw fibers and electrospun webs were sealed in Al crucibles and subjected to a heating temperature program in a temperature range from 10 to 210 °C, respectively. The conditions of the temperature program were as follows: heating rate: 2 °C/min; modulation period: 60 s; modulation amplitude: 0.5 °C. TMDSC was performed in dry N_2_ as a purge gas at a flow rate of 50 mL/min. Crystallinities of the samples were analyzed from the crystal melting endotherm, which was obtained by subtracting the exothermic heat of cold crystallization from the endothermic heat of melting in the total heat flow. A heat of fusion for 100% crystallinity of 106 J/g was employed for the calculation of crystallinity [37].

### 2.8. WAXD

WAXD (MiniFlex 600, Rigaku Co., Tokyo, Japan) was conducted using Ni-filtered CuKα radiation of wavelength 1.54056 Å generated at a voltage of 40 kV and a current of 15 mA. WAXD data were acquired using a charge-coupled detector in a diffraction angle (2θ) range of 10–55° at a scanning speed of 0.2°/min and with a step size of 0.01°. Prior to WAXD analysis, the BC sample was ground to finer particles, whereas the raw fiber samples were cut into extremely small powder-like pieces for LES to eliminate the effect of orientation. The BC and powdered fiber samples were placed between thin amorphous films for WAXD.

In addition, WAXD θ–2θ intensity distribution measurements of the bundles of raw PLLA and BC/PLLA fibers, arranged in parallel and stacked PLLA and BC/PLLA films with a thickness of 111 μm, were performed at azimuthal angles of 0 and 90° to acquire information regarding the degree of orientation.

### 2.9. Ultraviolet-Visible (UV-Vis) Spectroscopy

UV-Vis spectra were achieved using a spectrophotometer (V-670, JASCO Co., Hachiojishi, Tokyo, Japan) operating in the transmission mode and equipped with an integrating sphere. Measurement was conducted in the transmission mode under the following conditions: wavelength range: 200–850 nm; scanning speed: 400 nm/min; sampling interval: 0.5 nm. Area densities of BC, PLLA films, and BC/PLLA films were approximately 25 mg/cm^2^, whereas those of the electrospun webs were approximately 2.6 mg/cm^2^.

### 2.10. Polarizing Microscope

To investigate the birefringence of the fiber samples, a polarizing microscope (BX53-P, Olympus Co., Ltd., Tokyo, Japan) equipped with a polarizing filter and a Berek compensator was used to measure the optical retardation. Before observation, the raw PLLA fiber sample was placed between a glass slide and a cover glass using an immersion liquid. Birefringence was calculated using Equation (5):

(5) $$\Delta n=\frac{R}{d}$$

where Δn is the birefringence, R is the optical retardation, and d is the fiber diameter.

## 3. Results and Discussion

### 3.1. Thinning Behavior of the Fiber near the Nozzle during LES 

**Figure 2:**
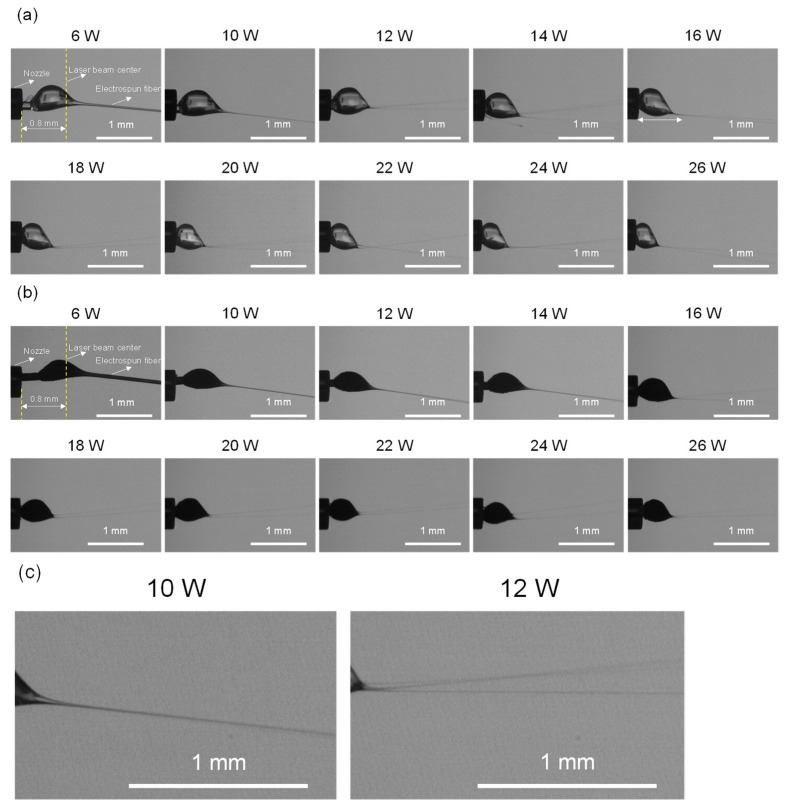
Images of the thinning behaviors (**a**) poly-(L-lactide) (PLLA), (**b**) bamboo charcoal (BC)/PLLA fibers near the nozzle at laser powers of 6–26 W. (**c**) Enlarged images for the PLLA spinning at laser powers of 10 W (single jet) and 12 W (multiple jet) are also shown.

Thinning behaviors of PLLA and BC/PLLA fibers during LES at different laser powers were examined by observing the fibers near the tip of the nozzle where the CO_2_ laser was irradiated. Images showing the deformation of the molten raw fiber and its thinning behavior were obtained, as shown in Figure 2. It was speculated that the initial increase in diameter, that is, the formation of a spherical droplet, was attributable to the cohesive force induced by surface tension and the sufficient reduction in viscosity caused by laser heating [38]. Subsequent thinning of the fiber was induced by electrostatic forces [38]. For both PLLA and BC/PLLA fibers, the size of the spherical droplet decreased and the starting point of the formation of thin fibers shifted upstream with an increase in laser power, whereas the droplet was prolonged and became smaller with the addition of BC.

Note that in the low laser power region, at a laser power lower than 12 W for PLLA samples and lower than 16 W for BC/PLLA samples, a single thin fiber was ejected from the droplet, whereas multiple fibers were ejected when the laser power was increased. The mechanism for the appearance of multiple fiber jets can be explained as follows: with an increase in laser power, the temperature increased, whereas polymer viscosity and surface tension decreased; as the reduction in viscosity was more significant, additional surface area was required to accommodate the repulsive charges [38]. For PLLA samples, multiple fibers started to continuously appear at a laser power of ≥12 W. In the case of BC/PLLA samples, intermittent ejection of multiple fibers was observed at a laser power of ≥16 W.

**Figure 3:**
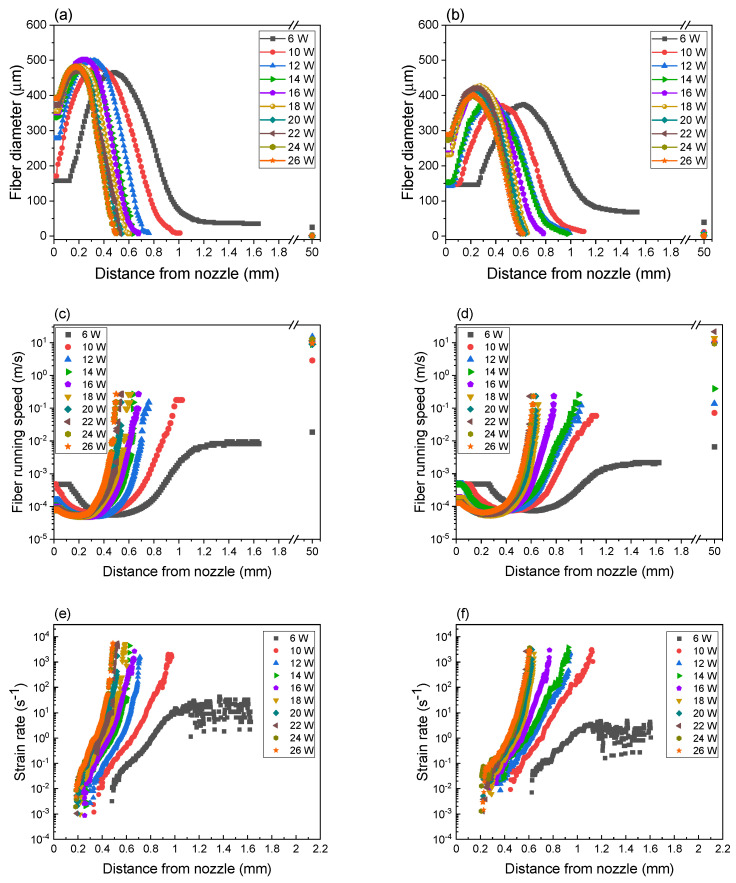
Diameter profiles of fibers in (**a**) PLLA and (**b**) BC/PLLA webs, running speeds of fibers in (**c**) PLLA and (**d**) BC/PLLA webs, and strain rate profiles of fibers in (**e**) PLLA and (**f**) BC/PLLA webs at laser powers of 6–26 W.

Fiber diameter profiles during the formation of PLLA and BC/PLLA webs were investigated from the obtained images, as shown in Figure 3a,b, respectively. Variations in fiber running speed along the spinline analyzed from the fiber diameter profiles of PLLA and BC/PLLA webs are shown in Figure 3c,d, respectively, and the strain rate profiles obtained by the derivation of fiber running speed at each point are depicted in Figure 3e,f, respectively. In Figure 3a–d, the average diameters of the as-spun fibers in the webs obtained from the SEM images and corresponding fiber running speeds are also plotted at 50 mm, where the fibers reached the collector.

As shown in Figure 3a,b, the fibers in PLLA and BC/PLLA webs exhibited similar fiber diameter profiles, that is, the fiber diameter initially increased and then decreased to extremely low values with an increase in the distance from the nozzle. As stated previously, regarding the diameter profile shown in Figure 3, it was speculated that the initial increase in diameter and the subsequent thinning of the fiber were attributable to the cohesive force and electrostatic force, respectively [38]. When the laser powers of less than 10 and 16 W were used for the preparation of PLLA and BC/PLLA webs, respectively, values of the raw fibers were maintained up to a certain distance from the nozzle, whereas the fiber diameter of the fibers at the tip of the nozzle gradually increased with an increase in laser power. 

The swelling zone, that is, the zone where the fiber diameter increases, moved upstream with an increase in laser power for both samples. Compared to the PLLA fibers, the BC/PLLA fibers exhibited smaller maximum diameter values, which were not significantly affected by the laser power. For the BC/PLLA fibers, the positions of maximum diameter were located downstream when compared with those for the PLLA fibers. In addition, the BC/PLLA fibers were mildly attenuated downstream as compared to the PLLA fibers.

In the cases of both PLLA and BC/PLLA fibers, the fiber running speed initially decreased with an increase in fiber diameter, and then sharply increased owing to the steep reduction in diameter (Figure 3c,d, respectively). With an increase in laser power, the fiber running speed and strain rate (Figure 3e,f) demonstrated a distinctly steeper increase at positions closer to the nozzle. Compared with those of the PLLA fibers, the fiber running speed and strain rate increased more slowly for the BC/PLLA fibers at a position further away from the nozzle. In the case of PLLA fibers, the maximum running speed and strain rate values achieved from the acquired image reached approximately 0.27 m/s and 5700 s^−1^, respectively. In contrast, the maximum running speed and strain rate values for the BC/PLLA fibers were approximately 0.23 m/s and 3800 s^−1^, respectively, which were smaller than those of PLLA fibers. Although the fiber running speed of 0.23–0.27 m/s, i.e., 13.8–16.2 m/min, in LES was low when compared with that of several hundred meters per minute in ordinary melt spinning, the maximum strain rate was extremely high, even higher than those evaluated for the neck-like deformation during high-speed melt spinning [39]. In addition, the maximum running speed was low but the maximum strain rate was high as compared with the behavior of jet near the Tayler cone apex during the electrospinning for poly(N-isopropylacrylamide) in dimethylformamide solvent [40]. 

**Figure 4:**
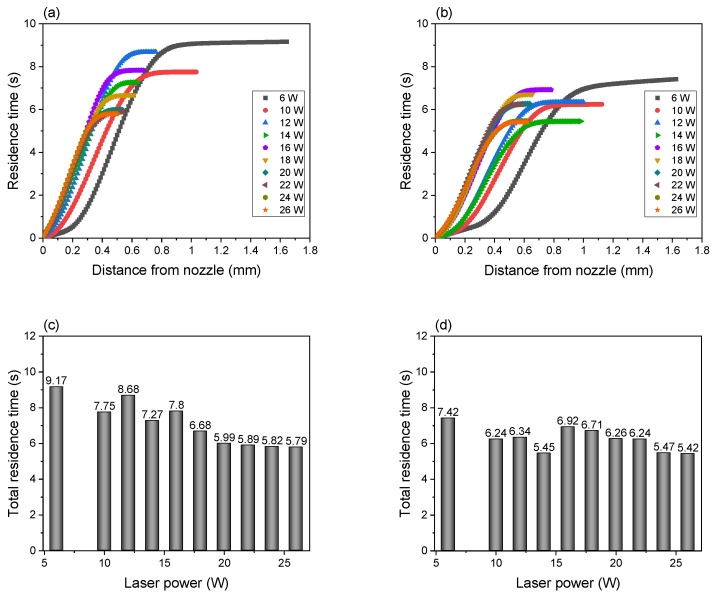
Residence time of (**a**) PLLA and (**b**) BC/PLLA fibers and total residence time of (**c**) PLLA and (**d**) BC/PLLA fibers near the nozzle at laser powers of 6–26 W.

By integrating the reciprocal of the fiber running speed profile along the spinline, the residence times of the PLLA and BC/PLLA fibers during LES were analyzed, as shown in Figure 4a,b, respectively. The relationship between laser power and total residence time was also plotted, as shown in Figure 4c,d. The residence time steeply increased with an increase in the diameter of the swelling zone. The residence time of PLLA fibers was shorter at 10 W as compared to that at 6 W. When the laser power was increased to 12 W, where multiple fiber jets started to be ejected, the residence time increased similarly with an increase in distance from the nozzle; however, the total residence time decreased with an increase in laser power. This tendency can be more clearly seen for BC/PLLA fibers. At a laser power of 6–14 W, where a single fiber jet was ejected, the total residence time decreased with an increase in laser power. For a laser power higher than 16 W, where multiple fiber jets were ejected, the residence time increased in a similar manner with an increase in distance from nozzle, and the total residence time decreased with an increase in laser power.

Note that the relationship between laser power and total residence time showed different trends depending on whether a single fiber jet or multiple fiber jets were ejected from the swelling zone. The PLLA and BC/PLLA fibers remained in the swelling zones for approximately 5.8–9.2 and 5.4–7.4 s, respectively. The shorter residence time of the BC/PLLA fibers in the region of low laser power may have originated from their higher absorbance. 

**Figure 5:**
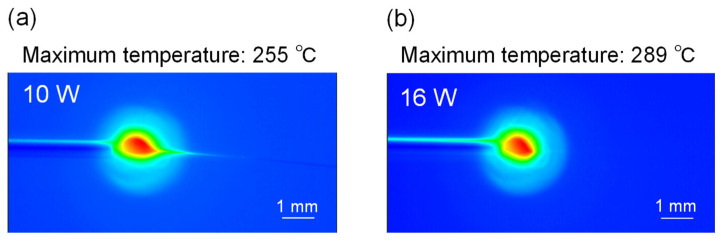
Infrared images of PLLA fibers near the nozzle during LES at laser powers of (**a**) 10 and (**b**) 16 W.

Figure 5 shows the temperature distribution of the swelling zone of PLLA fibers at the laser powers of 10 and 16 W. Measurement at higher laser powers was not conducted because of the limitation of the infrared thermometer used. The fiber temperature increased from the nozzle toward the melting area (namely, the laser-irradiated zone). The maximum temperatures estimated by speculating an emissivity of 0.95 [41,42] for PLLA fibers were approximately 255 and 289 °C at 10 and 16 W, respectively. The temperature of the electrospun fiber jet could not be evaluated because the fiber was too thin to be detected by the thermometer. 

Mechanism for the formation of the swelling zone can be described as follows. Surface tension is one of the important factors in the formation of swelling zones. Under the steady-state condition of fiber formation, the volume of the swelling zone is supposed to be constant. In this case, the amount of the raw fiber entering the swelling zone and the amount of the fiber jet emerging from the swelling zone need to be balanced. In contrast, the size of the swelling zone may be determined by the energy balance between the viscous flow for the formation of a polymer sphere and the reduction in the surface area. With an increase in temperature, the reduction in viscosity was more prominent when compared with that of surface tension. This indicates that a larger swelling zone is formed with an increase in laser power. 

The addition of BC to PLLA may not be straightforward. The introduction of BC into PLLA increases the absorptivity and viscosity of PLLA. The former leads to a reduction in viscosity through an increase in temperature. The absorption coefficient at the CO_2_ laser wavelength was analyzed via FTIR spectra and was found to be 3.42 × 10^3^ m^−1^ for the raw BC/PLLA fibers, which was approximately three times larger than that of the raw PLLA fibers (1.37 × 10^3^ m^−1^).

Another important factor in the formation of swelling zones is the effect of the viscosity of the polymer melt on the formation of a fiber jet at a certain applied voltage. Jet formation is governed by surface charge density and surface tension [38] and becomes easier with an increase in temperature, that is, with a reduction in viscosity. In this case, higher laser power may lead to the reduction in the size of the swelling zone and/or the ejection of multiple fiber jets. In the research on electrodynamic spraying of liquids, Hayati et al. reported that the stable single jets are only produced with optimal conducting liquid states when the appropriate voltage is applied, while the multiple jets occur with higher applied electric potential [43]. Note that the current measured at the collector was approximately 5–6 μA for both the PLLA and BC/PLLA fibers. The laser power and the addition of BC did not have a considerable effect on the measured current.

### 3.2. Morphologies and Diameter of Fiber Samples

**Figure 6:**
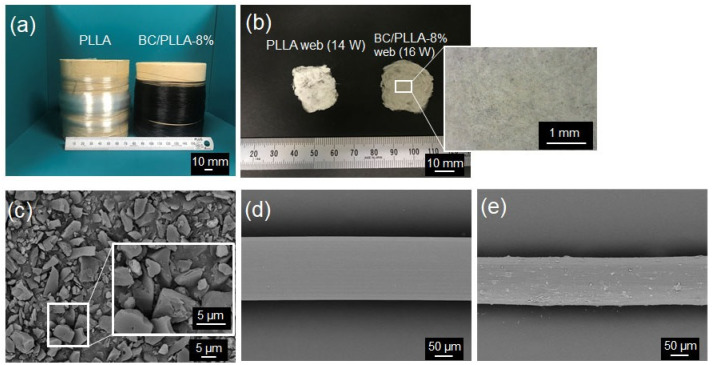
Images of (**a**) raw PLLA and BC/PLLA fibers and (**b**) electrospun PLLA (14 W) and BC/PLLA (16 W) webs and scanning electron microscopy (SEM) images of (**c**) BC, (**d**) raw PLLA fibers, and (**e**) raw BC/PLLA fibers.

Figure 6a,b show the images of raw PLLA and BC/PLLA fibers and PLLA and BC/PLLA webs, respectively, and Figure 6c–e depict the SEM images of BC, raw PLLA fibers, and raw BC/PLLA fibers, respectively. As observed from the images, the raw PLLA fibers are transparent, whereas the BC/PLLA fibers are black. The PLLA web is white, whereas the BC/PLLA web is gray, suggesting the existence of black BC particles in the BC/PLLA web. SEM images indicated that BC particles with a mean size of approximately 3–4 μm were extensively distributed in the PLLA matrix. The PLLA fiber had a smooth surface, whereas the raw BC/PLLA fiber had a rough surface because of the presence of BC particles.

**Figure 7:**
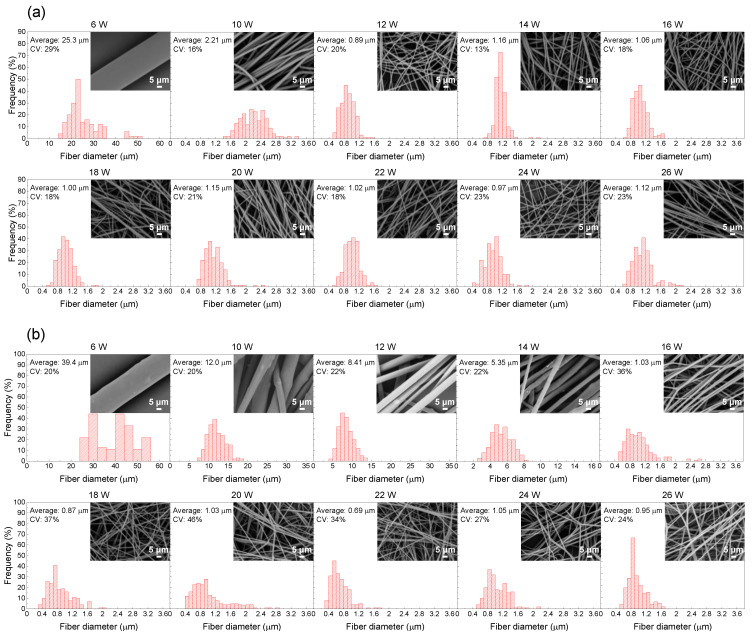
Diameter distributions: (**a**) PLLA and (**b**) BC/PLLA fibers in the electrospun webs fabricated at laser powers of 6–26 W.

Figure 7a,b show diameter distributions of the PLLA and BC/PLLA fibers, respectively, in the webs prepared at various laser powers. The average and CV values of the diameter distribution are shown in this figure. Electrospun webs with a relatively uniform diameter distribution and with the CV values of 13–23 and 20–46% were obtained for the PLLA and BC/PLLA fibers, respectively.

Insets in the SEM images of the PLLA and BC/PLLA webs show the respective frequency diagrams. The fibers in the PLLA webs had smooth surfaces at all laser powers. Nevertheless, the fibers in the BC/PLLA webs prepared at the laser powers of 6–14 W had rough surfaces due to the existence of BC particles. In contrast, the fibers in the BC/PLLA webs fabricated at the laser powers of more than 16 W exhibited smooth surfaces similar to those in the case of PLLA webs. This implied that although most of the BC particles remained in the web, they were excluded from the fibers. 

**Figure 8:**
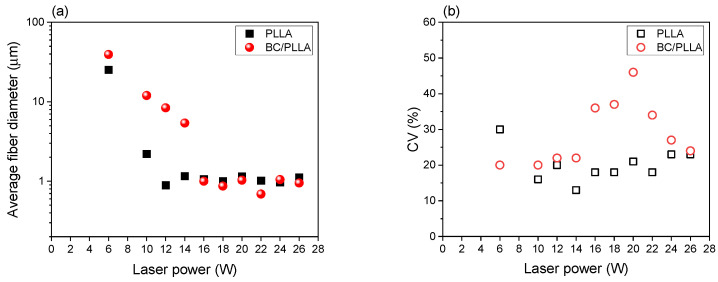
(**a**) Variations in the average fiber diameter and (**b**) coefficient of variation (CV) with laser power for the electrospun PLLA and BC/PLLA webs.

Figure 8 shows the variations in the average fiber diameter and CV with respect to laser power for the PLLA and BC/PLLA webs. When the laser power was increased from 6 W to 12 W and 16 W for PLLA and BC/PLLA webs, respectively, the average fiber diameter decreased. In this laser power range, the average fiber diameters for the BC/PLLA webs were larger than those for the PLLA webs. In the higher laser power range, which was higher than 12 and 16 W for the PLLA and BC/PLLA webs, respectively, corresponding to the laser power used for the formation of multiple fiber jets during LES, the average fiber diameter was almost constant, and ultrafine fiber webs with an average diameter of approximately 1 μm were obtained. The variation tendency of CV with respect to laser power was not clear; the BC/PLLA webs demonstrated higher CV, that is, a wider diameter distribution, specifically in the region of high laser power.

Based on a comprehensive investigation of the fiber diameter, the CV, and the SEM images of fibers in the webs, laser powers of 14 and 16 W were selected for the preparation of large amounts of PLLA and BC/PLLA webs, respectively, for further analysis of the characteristics of fibers in these webs. The average fiber diameter values for the PLLA and BC/PLLA webs were 1.16 and 1.03 μm, respectively.

### 3.3. Birefringence Analysis

**Figure 9:**
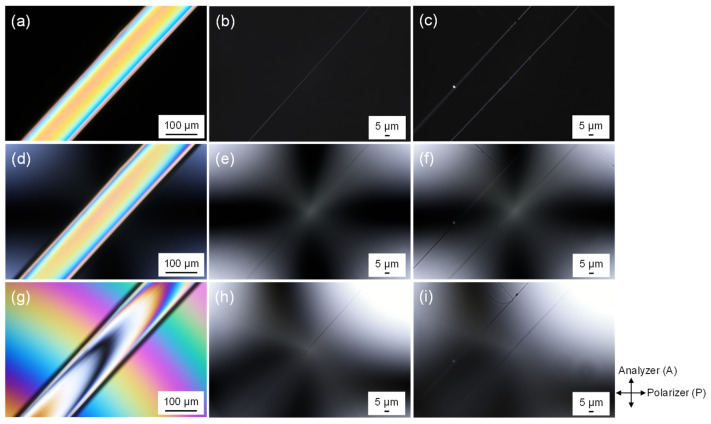
Images obtained under cross-polarization conditions for the (**a**) raw PLLA fiber, (**b**) electrospun PLLA web (14 W), and (**c**) electrospun BC/PLLA web (16 W); images acquired under cross-polarization conditions using the Bereck compensator without optical retardation for the (**d**) raw PLLA fiber, (**e**) electrospun PLLA web (14 W), and (**f**) electrospun BC/PLLA web (16 W); and images achieved under cross-polarization conditions with the Bereck compensator and optical retardation for the (**g**) raw PLLA fiber, (**h**) electrospun PLLA web (14 W), and (**i**) electrospun BC/PLLA web (16 W).

Images of the raw PLLA fiber and the fibers in the PLLA and BC/PLLA webs obtained using a polarizing microscope are shown in Figure 9. The images were acquired in the cross-nicol mode with and without the insertion of a Berek compensator. In general, the bright images of fibers obtained without the insertion of the compensator indicate that the fibers had a certain level of optical anisotropy, that is, birefringence.

When the compensator was used for the raw PLLA fiber, mean optical retardation values of approximately 0 and 560 nm were added. From the images, optical retardation of the raw fibers was determined to be approximately 560 nm. For the images of the fibers in the PLLA and BC/PLLA webs obtained using a polarizing microscope with the insertion of the compensator, as shown in Figure 9, the mean optical retardation values of approximately 0 and 23 nm were added. Without the addition of optical retardation, the fibers at the center of the image were faintly illuminated, whereas after adding an optical retardation of approximately 23 nm, the highest contrast of brightness between the background and fiber was obtained. This result indicates that the fiber had an optical retardation of approximately 23 nm. Birefringence of the observed fibers was calculated by dividing the optical retardation by the fiber diameter.

**Figure 10:**
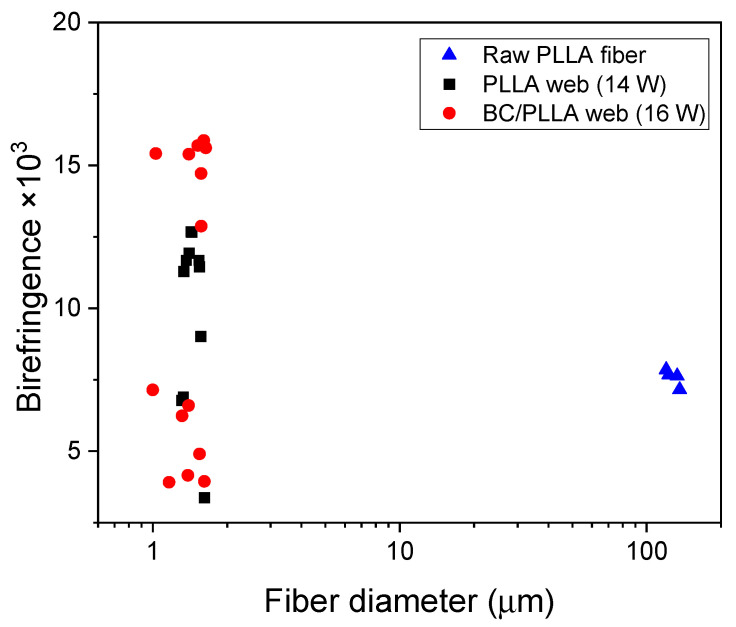
Correlation between the fiber diameter and birefringence of a raw PLLA fiber, an electrospun PLLA (14 W) web, and an electrospun BC/PLLA (16 W) web.

Birefringence values of the raw PLLA fiber and fibers in the PLLA and BC/PLLA webs were plotted against the fiber diameter (Figure 10). Birefringence of the raw PLLA fiber was approximately 7.6 × 10^−3^, implying that the fiber had a medium level of molecular orientation when compared with those of the high-speed spun fibers [44]. Birefringence of the raw BC/PLLA fiber could not be measured because transmittance of the light was extremely low. A wide distribution of birefringence was found for the fibers in the web samples, where a bimodal distribution with low- and high-birefringence fibers was noticed. The average birefringence values of the fibers in the PLLA and BC/PLLA webs were 7.4 and 9.7 × 10^−3^, respectively. 

### 3.4. WAXD Analysis

**Figure 11:**
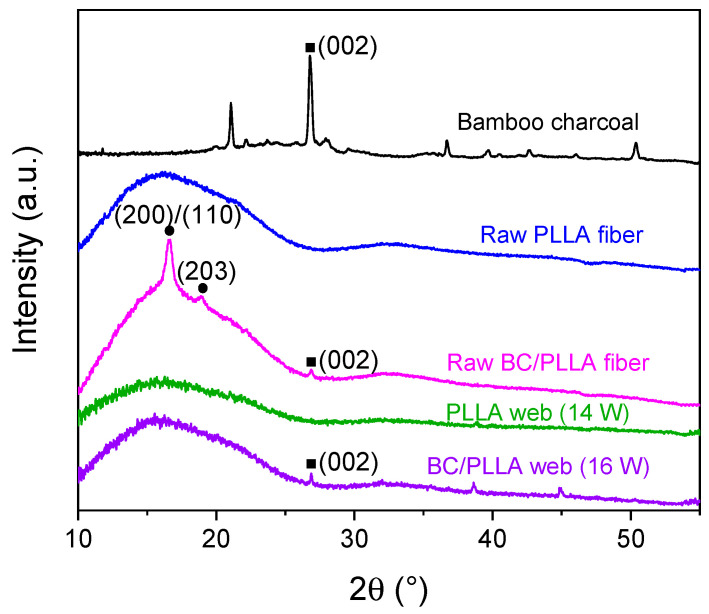
X-ray diffraction profiles of BC, raw PLLA and BC/PLLA fibers, and electrospun PLLA (14 W) and BC/PLLA (16 W) webs.

WAXD intensity profiles of BC powder, raw PLLA and BC/PLLA fibers, and PLLA and BC/PLLA webs, are presented in Figure 11. For the raw fibers and webs, the fibers were randomly oriented in the specimen for WAXD. The diffraction peaks of BC at 2θ ≈ 27° corresponded to the (002) crystal plane of the hexagonal crystal structure of graphite [45,46]. The raw PLLA fibers showed only an amorphous halo. Moreover, the peaks of the raw BC/PLLA fibers at 2θ ≈ 16.7 and 19° were assigned to the (200)/(110) and (203) crystal planes of PLLA [47], respectively, whereas the peak at 27° was attributed to the (002) crystal planes of graphite in BC. This result suggested that the addition of BC enhanced the crystallization of PLLA in the raw fibers.

In the WAXD pattern of BC/PLLA webs, diffraction peaks were noticed at 2θ ≈ 27°, which corresponded to the (002) plane of graphite in BC; nevertheless, distinct diffraction peaks of PLLA were not observed in the patterns of both the PLLA and BC/PLLA webs. 

**Figure 12:**
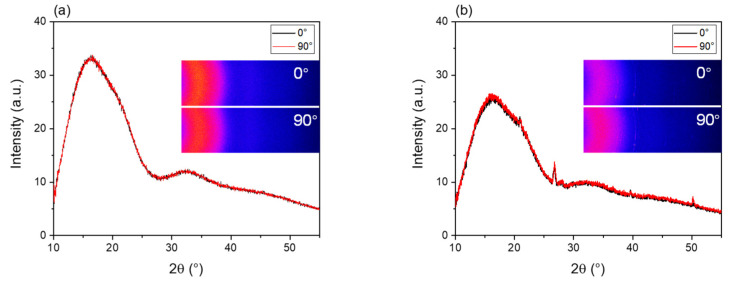
Wide-angle X-ray diffraction (WAXD) profiles and two-dimensional patterns at azimuthal angles of 0 and 90° for (**a**) PLLA and (**b**) BC/PLLA films.

WAXD intensity profiles of the PLLA and BC/PLLA films at azimuthal angles of 0 and 90° are shown in Figure 12a,b, respectively. Moreover, two-dimensional (2D) intensity diagrams near the equator and meridian, that is, at the azimuthal angles of 0 and 90°, respectively, are also shown in the insets of Figure 12. The WAXD profiles achieved at 0 and 90° are almost identical, indicating the presence of an isotropic structure in the films. The (002) peak of graphite in BC at a 2θ of approximately 27° was observed in the profile of the BC/PLLA film. Crystalline reflections from PLLA were not detected in the profiles of both PLLA and BC/PLLA films, implying that the PLLA matrix was in an amorphous state.

**Figure 13:**
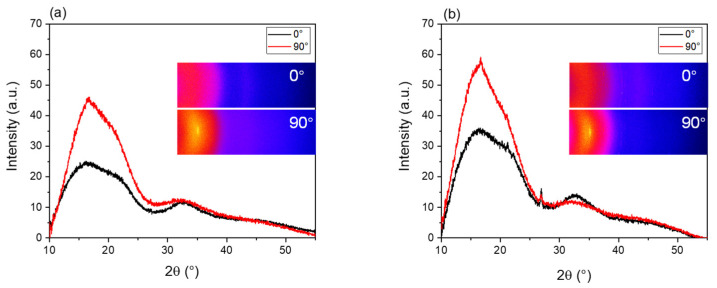
WAXD profiles at the azimuthal angles of 0 and 90° for raw (**a**) PLLA and (**b**) BC/PLLA fibers.

WAXD intensity profiles for the bundles of raw PLLA and BC/PLLA fibers at the azimuthal angles of 0 and 90° are depicted in Figure 13a,b, respectively. Two-dimensional intensity diagrams at the azimuthal angles of 0 and 90° are also shown in the insets of Figure 13. In the 2D diagrams, the intensity was higher at 90°, and weak crystalline reflection was observed for both the PLLA and BC/PLLA fibers. Accordingly, in the intensity profiles, the intensity of the broad amorphous halo in the 2θ range of 10–25° was substantially higher at 90° than that at 0°. In addition, a small peak related to the (200)/(110) plane of the α-form crystal of PLLA at 2θ = 16.7° was detected in the intensity profile of 90° for both the PLLA and BC/PLLA fibers, and the crystalline reflection was slightly stronger for the BC/PLLA fibers. These results indicate the orientation of the molecular chain in the amorphous phase and that of the crystalline *c*-axis along the fiber axis. The higher tendency of crystallization for the BC/PLLA fibers may be ascribed to the role of BC as a nucleating agent. In contrast, for the raw BC/PLLA fibers, the peak of the (002) plane of graphite in BC was noticed at a 2θ of approximately 27° in the intensity profiles for both 0 and 90°.

### 3.5. TMDSC 

**Figure 14:**
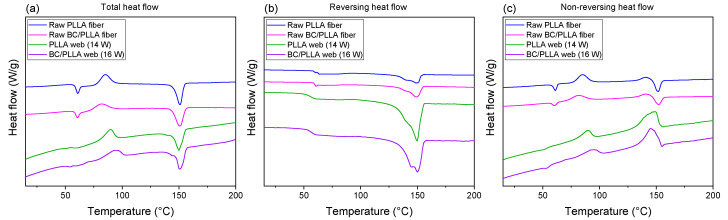
(**a**) Total heat flow curves, (**b**) reversing heat flow curves, and (**c**) non-reversing heat flow curves of raw PLLA and BC/PLLA fibers and electrospun PLLA (14 W) and BC/PLLA (16 W) webs determined by temperature-modulated differential scanning calorimetry (TMDSC).

TMDSC thermograms of the first and second heating processes of raw fibers and web samples are depicted in Figure 14. Glass transition temperatures (*T_g_*), peak temperatures of cold crystallization and melting (*T_c_* and *T_m_*, respectively), and crystallinity (*X_c_*), evaluated from the TMDSC thermograms, are presented in Table 1. 

Enthalpy relaxation was clearly observed in the total and non-reversing heat flows (THF and NRHF, respectively) along with glass transition in the reversing heat flow (RHF) for the raw fibers; in contrast, only glass transition was detected in the THF and RHF for the electrospun webs. *T_g_* values evaluated from the RHF for the raw fibers and webs were approximately 59 and 56 °C, respectively.

During the THF and NRHF of the raw PLLA and BC/PLLA fibers, an exothermic peak of *T_c_* appeared at ca. 85 and 82 °C, respectively. The *T_c_* peak of the raw BC/PLLA fiber shifted to a lower temperature when compared with that of the raw PLLA fiber, indicating that the crystallization rate of the raw BC/PLLA fiber was accelerated, probably due to higher molecular orientation in this fiber during the spinning process. 

Compared with those for the raw fibers, the *T_c_* peaks during the THF and NRHF of the PLLA and BC/PLLA web samples appeared at higher temperatures of approximately 88 and 95 °C, respectively, demonstrating lower molecular orientations in the webs than those in the raw fibers. The *T_c_* peaks of the web samples were significantly broader, with a long tail toward lower temperatures. This may be caused by the wide distribution of molecular orientations, as indicated by the birefringence results shown in Figure 10 [48].

Regarding the melting behavior, a single endothermic peak with starting and peak temperatures of 140 and 150 °C, respectively, was observed for the raw fibers in the THF, whereas melting started at lower temperatures of approximately 130 °C for the web samples. Nevertheless, in the RHF, even for the raw fibers, melting began at lower temperatures when compared with that in the THF. Melting of the web samples started at lower temperatures in both the THF and the RHF as compared to that of the raw fibers in the THF. Furthermore, the melting peaks of the web samples in the RHF were substantially larger than those of the raw fibers. 

Differences between the melting behaviors of raw fibers and web samples were more distinct in the NRHF. For the raw fibers, a small exothermic peak was obtained at approximately 140 °C, followed by a clear endothermic peak at approximately 150 °C. Simultaneous appearance of endothermic heat flow in the RHF and exothermic heat flow in the NRHF indicated the occurrence of melting and recrystallization. Instead, the unusual appearance of an endothermic peak before total melting in the NRHF suggested the existence of highly ordered crystals. For the web samples, only exothermic heat flow was observed during the NRHF, implying that the degree of order of the crystals was lower than that in the case of raw fibers. Larger endothermic and exothermic heat flows during the RHF and NRHF, respectively, for the web samples may suggest higher mobility of the molecules in these samples. That is, the mobility of chain molecules was constrained in the raw fibers. 

**Figure 15:**
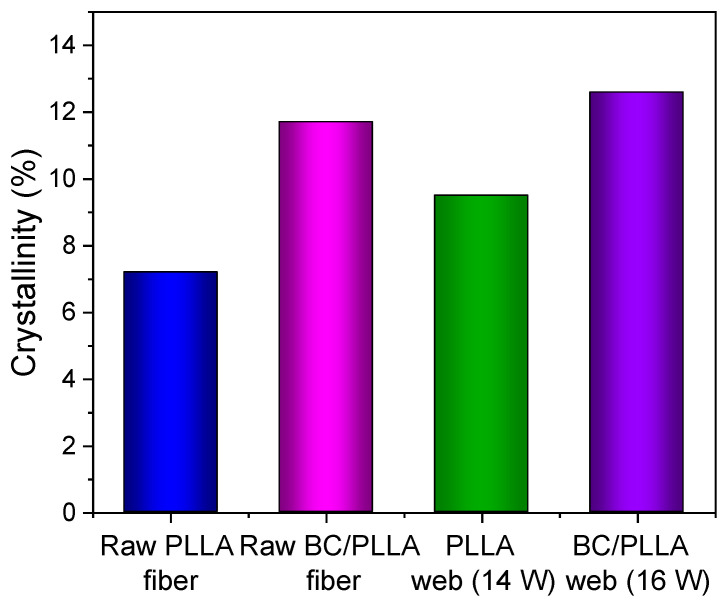
Crystallinities of raw PLLA and BC/PLLA fibers and electrospun PLLA (14 W) and BC/PLLA (16 W) webs calculated from total heat flow curves.

Crystallinity of the raw fibers and web samples was estimated from the THF during heating, as shown in Figure 15. In particular, the crystallinities of the web samples were higher than those of the raw fibers, and the crystallinities of the BC/PLLA samples were higher than those of the PLLA samples. WAXD indicated that the raw fibers and web samples were generally in amorphous states, except for the formation of only a small amount of crystals for the raw fibers. In contrast, the crystallinities of the oriented amorphous samples determined via TMDSC by subtracting the exothermic heat of cold crystallization from the endothermic heat of melting do not become zero and tend to increase with an increase in molecular orientation [44,48]. The higher crystallinity of the web samples may have originated from the existence of fibers with high birefringence in them, as shown in the birefringence versus fiber diameter diagram in Figure 10. Specifically, the smaller *T_c_* peak of the fibers with high molecular orientations in the web may result in higher crystallinity, and the higher crystallinity of the BC/PLLA web than that of the PLLA web can also be attributed to the presence of fibers with higher birefringence in the BC/PLLA web. 

### 3.6. UV-Vis Shielding

**Figure 16:**
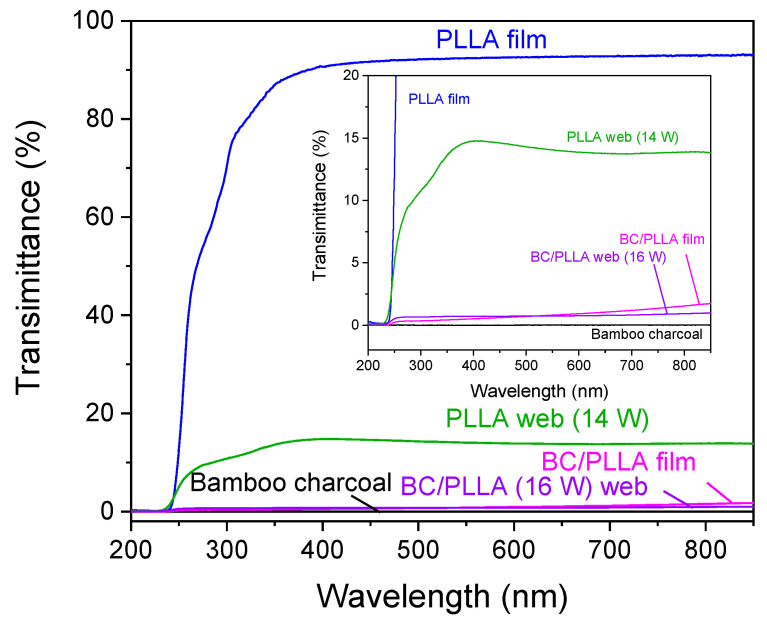
Ultraviolet–visible transmission spectra of PLLA and BC/PLLA films and electrospun PLLA (14 W) and BC/PLLA (16 W) webs.

High transparency of PLLA to UV light is an issue that needs to be overcome to avoid the degradation of PLLA by UV in its applications [22]. Generally, several methods are used to improve the UV-protection ability of PLLA. Adding a dark pigment to PLLA to decrease the transmittance of UV light is one way to realize the abovementioned goal [49]. Therefore, in our study, BC was introduced into PLLA, and the corresponding web samples exhibited high UV-barrier properties. Accordingly, the light-barrier properties of the BC/PLLA web were evaluated by UV-Vis transmission spectroscopy in the wavelength range of 200–850 nm. As shown in Figure 16, of the BC powder with an area density of 25 mg/cm^2^, approximately 0% was at wavelengths below 400 nm (UV light wavelength) and above 400 nm (Vis light wavelength), indicating high UV-shielding efficiency of BC. Compared with those of the PLLA film and web, the transmittance values of the BC/PLLA film and web were lower over the entire measurement range. Transmittance of PLLA at a wavelength of 300 nm decreased from 70 (for the PLLA film) and 11% (for the PLLA web) to 0.3 (for the BC/PLLA film) and 0.7% (for the BC/PLLA web) upon the introduction of BC. When the wavelength was higher than 400 nm, the BC/PLLA film and web maintained their low transmittance of approximately 1 and 6%, respectively. These results demonstrated that the addition of BC improved the UV-shielding efficiency of PLLA as expected, even for the webs prepared through melt electrospinning.

## 4. Conclusions

In this study, ultrafine fiber webs of PLLA and BC/PLLA containing 0 and 8 wt% BC, respectively, were prepared by LES, and the effects of the addition of BC to PLLA on the structures and properties of the raw fibers for LES, and the resulting webs, were investigated. PLLA and BC/PLLA webs consisting of ultrafine fibers of fairly uniform diameters (average fiber diameter of approximately 1 μm, and CV of 13–23% and 20–46% for the PLLA and BC/PLLA fibers, respectively) were obtained at laser powers higher than 12 and 16 W, respectively. Observation of the spinline revealed that a single fiber jet was ejected in the low laser power region, whereas multiple fiber jets started to form when the laser power exceeded a certain value. The critical laser power for the formation of multiple fiber jets was higher for the BC/PLLA fibers. In contrast, the fiber diameter decreased with an increase in laser power for the single fiber jet ejection, whereas fiber diameter remained almost constant for the multiple fiber jet ejection, and a minimum fiber diameter of approximately 1 μm was obtained for both the PLLA and BC/PLLA webs. The size of the spherical droplet of molten material decreased with an increase in laser power, demonstrating different trends for single and multiple fiber jet ejections as the size was larger for the multiple fiber jet ejection. The birefringence of the laser-electrospun fibers in the webs showed a wide distribution of low and high values; nevertheless, the fiber diameter was considerably uniform. WAXD implied that a small amount of highly oriented crystals existed in the raw fibers, whereas the fibers in the webs were in amorphous states; however, some fibers showed high molecular orientations. For the BC/PLLA webs, the diffraction peaks of graphite indicated that BC was still present in these webs. In the TMDSC thermograms of the web samples, a broad cold crystallization peak with a long tail toward lower temperatures appeared in the total and non-reversing heat flows. With respect to the melting behavior, although the raw fibers exhibited exothermic and endothermic peaks consecutively in the non-reversing heat flow, only exothermic heat flow was observed for the webs in the non-reversing heat flow, suggesting that the degrees of order of the crystals in webs was lower than those in the cases of the raw fibers. Larger endothermic and exothermic heat flows for the web samples in the reversing and non-reversing heat flows, respectively, when compared with those in the cases of the raw fibers, may also imply higher mobility of molecules in the webs. As observed from the UV-Vis transmisson spectra, transmittance at a wavelength of 300 nm for the webs with area densities of 2.6 mg/cm^2^ decreased from 11% for the PLLA webs to 0.7% for the BC/PLLA webs. When the wavelength was higher than 400 nm, the BC/PLLA webs maintained a low transmittance of approximately 6%. These results demonstrated that the presence of BC improved the UV-shielding efficiency of PLLA fibers as expected, even for the webs prepared through LES.

## Figures and Tables

**Table 1:** Temperature-modulated differential scanning calorimetry results for raw poly-(L-lactide) (PLLA) and bamboo charcoal (BC)/PLLA fibers and electrospun PLLA (14 W) and BC/PLLA (16 W) webs.

Samples	*T_g Reversing_* (°C)	*T_c_* (°C)	*T_m_*_1_ (°C)	*T_m_*_2_ (°C)	*X_c_* (%)
Raw PLLA fiber	59.4	85.3	-	150.5	7.2
Raw BC/PLLA fiber	59.7	82.1	-	150.5	11.7
PLLA web (14 W)	55.5	89.9	139.9	150.0	9.5
BC/PLLA web (16 W)	56.5	95.2	145.0	150.5	12.6

## Data Availability

The data that support the findings of this study are available from the corresponding author, upon reasonable request.

## Nomenclature

v	Fiber running speed in the spinline (m/s)
$v_{0}$	Feeding speed of the raw melt-spun fiber (m/s)
D	Diameter in the spinline (m)
$D_{0}$	Diameter of the raw melt-spun fiber (m)
$\dot{\varepsilon }$	Strain rate (s^−1^)
x	Distance from the nozzle (mm)
t(x)	Residence time (s)
A	Absorbances
K	Absorption coefficient (m^−1^)
T	Transmittance
$x_{t}$	Thickness of the film (m)
$A_{0}$	Effect of the surface reflection
Δn	Birefringence
R	Optical retardation (nm)
d	Fiber diameter (μm)

## References

[1] Park J.H., Rutledge G.C. (2018). Ultrafine high performance polyethylene fibers. J. Mater. Sci..

[2] Zhang Z., Ji D., He H., Ramakrishna S. (2021). Electrospun ultrafine fibers for advanced face masks. Mater. Sci. Eng. R Rep..

[3] Balakrishnan N.K., Koenig K., Seide G. (2020). The effect of dye and pigment concentrations on the diameter of melt-electrospun polylactic acid fibers. Polymers.

[4] Yu Y., Xiong S., Huang H., Zhao L., Nie K., Chen S., Xu J., Yin X., Wang H., Wang L. (2020). Fabrication and application of poly (phenylene sulfide) ultrafine fiber. React. Funct. Polym..

[5] Iordanskii A., Karpova S., Olkhov A., Borovikov P., Kildeeva N., Liu Y. (2019). Structure-morphology impact upon segmental dynamics and diffusion in the biodegradable ultrafine fibers of polyhydroxybutyrate-polylactide blends. Eur. Polym. J..

[6] Zapletalova T., Michielsen S., Pourdeyhimi B. (2006). Polyether based thermoplastic polyurethane melt blown nonwovens. J. Eng. Fibers Fabr..

[7] Zhang X., Lu Y. (2014). Centrifugal spinning: An alternative approach to fabricate nanofibers at high speed and low cost. Polym. Rev..

[8] Weitz R.T., Harnau L., Rauschenbach S., Burghard M., Kern K. (2008). Polymer nanofibers via nozzle-free centrifugal spinning. Nano Lett..

[9] Xue J., Wu T., Dai Y., Xia Y. (2019). Electrospinning and electrospun nanofibers: Methods, materials, and applications. Chem. Rev..

[10] Takasaki M., Nakashima K., Tsuruda R., Tokuda Y., Tanaka K., Kobayashi H. (2019). Drug release behavior of a drug-loaded polylactide nanofiber web prepared via laser-electrospinning. J. Macromol. Sci. B..

[11] Wang Y.B., Tian L., Zhu T.H., Mei J., Chen Z.Z., Yu D.G. (2021). Electrospun aspirin/Eudragit/lipid hybrid nanofibers for colon-targeted delivery using an energy-saving process. Chem. Res. Chin. Univ..

[12] Ponnamma D., Chamakh M.M., Alahzm A.M., Salim N., Hameed N., AlMaadeed M.A.A. (2020). Core-shell nanofibers of polyvinylidene fluoride-based nanocomposites as piezoelectric nanogenerators. Polymers.

[13] Wang M.L., Li D., Li J., Li S.Y., Chen Z., Yu D.G., Liu Z.P., Guo Z.H. (2020). Electrospun Janus zein–PVP nanofibers provide a two-stage controlled release of poorly water-soluble drugs. Mater. Des..

[14] Ding Y.F., Dou C.H., Chang S.Y., Xie Z.M., Yu D.G., Liu Y.N., Shao J. (2020). Core–shell eudragit s100 nanofibers prepared via triaxial electrospinning to provide a colon-targeted extended drug release. Polymers.

[15] Aidana Y., Wang Y., Li J., Chang S., Wang K., Yu D.G. (2021). Fast dissolution electrospun medicated nanofibers for effective delivery of poorly water-soluble drugs. Curr. Drug Deliv..

[16] Ostheller M.E., Balakrishnan N.K., Groten R., Seide G. (2021). Detailed process analysis of biobased polybutylene succinate microfibers produced by laboratory-scale melt electrospinning. Polymers.

[17] Kang S.X., Hou S.C., Chen X.W., Yu D.G., Wang L., Li X.Y., Williams G.R. (2020). Energy-saving electrospinning with a concentric teflon-core rod spinneret to create medicated nanofibers. Polymers.

[18] King W.E., Bowlin G.L. (2021). Near-field electrospinning and melt electrowriting of biomedical polymers—Progress and limitations. Polymers.

[19] Li X., Liu H., Wang J., Li C. (2012). Preparation and characterization of PLLA/nHA nonwoven mats via laser melt electrospinning. Mater. Lett..

[20] Takasaki M., Sugihara K., Ohkoshi Y., Fujii T., Shimizu H., Saito M. (2010). Thermoplastic polyurethane ultrafine fiber web fabricated by laser electrospinning. Sen’i Gakkaishi.

[21] Tokuda T., Tsuruda R., Hara T., Kobayashi H., Tanaka K., Takarada W., Kikutani T., Hinestroza J.P., Razal J.M., Takasaki M. (2020). Structure and Properties of Poly (ethylene terephthalate) Fiber Webs Prepared via Laser-Electrospinning and Subsequent Annealing Processes. Materials.

[22] Lizundia E., Vilas J.L., Sangroniz A., Etxeberria A. (2017). Light and gas barrier properties of PLLA/metallic nanoparticles composite films. Eur. Polym. J..

[23] Wang D.Y., Song Y.P., Lin L., Wang X.L., Wang Y.Z. (2011). A novel phosphorus-containing poly (lactic acid) toward its flame retardation. Polymer.

[24] Tertyshnaya Y., Podzorova M., Moskovskiy M. (2021). Impact of Water and UV Irradiation on Nonwoven Polylactide/Natural Rubber Fiber. Polymers.

[25] Zhou H., Green T.B., Joo Y.L. (2006). The thermal effects on electrospinning of polylactic acid melts. Polymer.

[26] Hong Y., Fujimoto K., Hashizume R., Guan J., Stankus J.J., Tobita K., Wagner W.R. (2008). Generating elastic, biodegradable polyurethane/poly (lactide-co-glycolide) fibrous sheets with controlled antibiotic release via two-stream electrospinning. Biomacromolecules.

[27] Athanasoulia I.G., Giachalis K., Korres D., Todorova N., Giannakopoulou T., Tarantili P.A., Trapalis C. (2020). Study of thermomechanical, structural and antibacterial properties of poly (lactic acid) reinforced with graphene oxide nanoparticles via melt mixing. Polym. Int..

[28] Ho M., Lau K., Wang H., Hui D. (2015). Improvement on the properties of polylactic acid (PLA) using bamboo charcoal particles. Compos. Part B Eng..

[29] Wang S., Zhang L., Semple K., Zhang M., Zhang W., Dai C. (2020). Development of Biodegradable Flame-Retardant Bamboo Charcoal Composites, Part II: Thermal Degradation, Gas Phase, and Elemental Analyses. Polymers.

[30] Lou C.W., Lin C.W., Lei C.H., Su K.H., Hsu C.H., Liu Z.H., Lin J.H. (2007). PET/PP blend with bamboo charcoal to produce functional composites. J. Mater. Process. Technol..

[31] Qian S., Yan W., Zhu S., Fontanillo Lopez C.A., Sheng K. (2018). Surface modification of bamboo-char and its reinforcement in PLA biocomposites. Polym. Compos..

[32] Wang S., Zhao L., Zhou H., Li W., Zhang W. (2020). Effect of Aluminum Hypophosphite on Flame Retardancy and Mechanical Property of Bamboo Charcoal/Polylactic Acid Composites. Mater. Rep..

[33] Zhu L., Zhou X., Liu Y.H., Fu Q. (2019). Highly sensitive, ultrastretchable strain sensors prepared by pumping hybrid fillers of carbon nanotubes/cellulose nanocrystal into electrospun polyurethane membranes. ACS Appl. Mater. Interfaces.

[34] Haroosh H.J., Chaudhary D.S., Dong Y. (2012). Electrospun PLA/PCL fibers with tubular nanoclay: Morphological and structural analysis. J. Appl. Polym. Sci..

[35] Guo Y.Q., Yang X.T., Ruan K.P., Kong J., Dong M.Y., Zhang J.X., Gu J.W., Guo Z.H. (2019). Reduced graphene oxide heterostructured silver nanoparticles significantly enhanced thermal conductivities in hot-pressed electrospun polyimide nanocomposites. ACS Appl. Mater. Inter..

[36] Takasaki M., Kengo M., Ohkoshi Y., Hirai T. (2015). Effects of Laser Beam Width on the Diameter and Molecular Weight of Laser-Electrospun Polylactide Fiber. Sen’i Gakkaishi.

[37] Zuza E., Ugartemendia J.M., Lopez A., Meaurio E., Lejardi A., Sarasua J. (2008). Glass transition behavior and dynamic fragility in polylactides containing mobile and rigid amorphous fractions. Polymer.

[38] Andrady A.L. (2008). Science and Technology of Polymer Nanofibers.

[39] Murase H., Yabuki K., Tashiro K., Kikutani T. (2016). Advancement of Fiber Science and Technology. High-Performance and Specialty Fibers: Concepts, Technology and Modern Applications of Man-Made Fibers for the Future.

[40] Wang C., Wang Y., Hashimoto T. (2016). Impact of entanglement density on solution electrospinning: A phenomenological model for fiber diameter. Macromolecules.

[41] Wijnen B., Sanders P., Pearce J.M. (2018). Improved model and experimental validation of deformation in fused filament fabrication of polylactic acid. Prog. Addit. Manuf..

[42] Zhao X., Guerrero F.R., Llorca J., Wang D.Y. (2016). New superefficiently flame-retardant bioplastic poly (lactic acid): Flammability, thermal decomposition behavior, and tensile properties. ACS. Sustain. Chem. Eng..

[43] Hayati I., Bailey A.I., Tadros T.F. (1987). Investigations into the mechanisms of electrohydrodynamic spraying of liquids: I. Effect of electric field and the environment on pendant drops and factors affecting the formation of stable jets and atomization. J. Colloid Interface Sci..

[44] Takasaki M., Ito H., Kikutani T. (2003). Structure Development of Polylactides with Various D-lactide Contents in the High-Speed Melt Spinning Process. J. Macromol. Sci. B.

[45] Seehra M.S., Geddam U.K., Schwegler-Berry D., Stefaniak A.B. (2015). Detection and quantification of 2H and 3R phases in commercial graphene-based materials. Carbon.

[46] Seehra M.S., Narang V., Geddam U.K., Stefaniak A.B. (2017). Correlation between X-ray diffraction and Raman spectra of 16 commercial graphene-based materials and their resulting classification. Carbon.

[47] Zhang J., Tashiro K., Tsuji H., Domb A.J. (2008). Disorder-to-order phase transition and multiple melting behavior of poly (L-lactide) investigated by simultaneous measurements of WAXD and DSC. Macromolecules.

[48] Murase Y., Nagai A., Nakajima T., Kajiwara K., McIntyre J.E. (1994). 2-Melt spinning. Advanced Fiber Spinning Technology.

[49] Evans P., Chowdhury M.J., Mathews B., Schmalzl K., Ayer S., Kiguchi M., Kataoka Y., Kutz M. (2005). Weathering and surface protection of wood. Handbook of Environmental Degradation of Materials.

